# Effect of CeO_2_ Size on Microstructure, Synthesis Mechanism and Refining Performance of Al-Ti-C Alloy

**DOI:** 10.3390/ma14226739

**Published:** 2021-11-09

**Authors:** Yanli Ma, Taili Chen, Lumin Gou, Wanwu Ding

**Affiliations:** 1 Technology Center, Jiuquan Iron and Steel (Group) Co., Ltd., Jiayuguan 735100, China; mayanli@jiugang.com; 2 School of Materials Science and Engineering, Lanzhou University of Technology, Lanzhou 730050, China; chen_taili@163.com (T.C.); goulumin@163.com (L.G.)

**Keywords:** CeO_2_ size, quenching experiment method, DSC, refining performance

## Abstract

The effects of CeO_2_ size on the microstructure and synthesis mechanism of Al-Ti-C alloy were investigated using a quenching experiment method. A scanning calorimetry experiment was used to investigate the synthesis mechanism of TiC, the aluminum melt in situ reaction was carried out to synthesize master alloys and its refining performance was estimated. The results show that the Al-Ti-C-Ce system is mainly composed of α-Al, Al_3_Ti, TiC and Ti_2_Al_20_Ce. The addition of CeO_2_ obviously speeds up the progress of the reaction, reduces the size of Al_3_Ti and TiC and lowers the formation temperature of second-phase particles. When the size of CeO_2_ is 2–4 μm, the promotion effect on the system is most obvious. The smaller the size of CeO_2_, the smaller the size of Al_3_Ti and TiC and the lower the formation temperature. Al-Ti-C-Ce master alloy has a significant refinement effect on commercial pure aluminum. When the CeO_2_ size is 2–4 μm, the grain size of commercial pure aluminum is refined to 227 μm by Al-Ti-C-Ce master alloy.

## 1. Introduction

A number of studies show that grain refiner for aluminum and its alloys can significantly improve the mechanical properties, casting properties, deformation processing properties and surface quality of materials [1,2].

Al-Ti-B master alloy has been the most widely used grain refiner and has been applied commercially [3]. However, many defects have been found in the application of Al-Ti-B master alloy thus limiting its development, i.e., a larger aggregation tendency of TiB_2_ in the melt, large size, and the possibility of Zr and Si poisoning [4,5]. In order to obtain a good refining efficiency, a series of novel master alloys have emerged, such as Al-Ti-C, Al-Ti-B-C and Al-Ti-C/B-RE master alloy [6,7,8,9]. Al-Ti-C master alloy especially has been extensively studied by researchers, and it has been found that it can be remedy certain defects of Al-Ti-B master alloy [10,11]. Unfortunately, the refining efficiency of Al-Ti-C is unstable, and the possible reasons for this are that the active of TiC is insufficient, TiC in the melt aggregates easily and the morphology of Al_3_Ti and TiC is easily affected by preparation conditions. For the most part, the wetting between Al and C is poor, and the formation of TiC is therefore difficult [12].

Ao et al. [13] found that La_2_O_3_ added to the Al-Ti-C-CuO system could promote the wettability of C and Al melt. The results of thermodynamic analysis from differential scanning calorimetry (DSC) experiments showed that La_2_O_3_ could promote metastable phase transfer into stable phases. Furthermore, the even distribution of TiC can directly influence the refining efficiency of Al-Ti-C. Wang L. D. et al. [14] analyzed the thermodynamics of the Al-Ti-C-Ce system using DSC experiments, and the result showed that rare earth oxide promoted the reaction and made the distribution of TiC even, forming a new Ti_2_Al_20_Ce phase. Moreover, CeO_2_ has a catalytic effect and can promote the reaction. Korotcenkov et al. [15] considered that the catalytic activity of CeO_2_ is closely related to its structure, morphology and size. The research of Auffan et al. [16] and Liu et al. [17] shows that CeO_2_ nanoparticles have a high specific surface area, and the smaller the size of CeO_2_ nanoparticles, the higher the specific surface area and the greater the activity. Preliminary studies have shown that when the content of CeO_2_ was 4 wt.%, it had a significant promoting effect on the Al-Ti-C system [18]. On this basis, it is necessary to further study the effect of CeO_2_ particle size on phase transition and microstructure transformation of Al-Ti-C system.

In this study, the aluminum melt in situ reaction was carried out to prepare Al-Ti-C-Ce master alloys. The influence of the size of CeO_2_ on the thermodynamic process, reaction products and microstructures of the prepared Al-Ti-C-Ce system were studied in detail through quenching experiments, and the reaction mechanism of the Al-Ti-C-Ce system is summarized in this paper. The goal of the abovementioned studies was to determine the influence mechanism on the microstructure and phase transformation with different sizes of CeO_2_ in the Al-Ti-C-Ce system based on thermodynamic analysis and DSC experiments.

## 2. Experiment

### 2.1. Quenching Experiments

Al powders (99.0 wt.%, 80–100 µm), Ti powders (99.0 wt.%, 45–65 µm) and C powders (99.0 wt.%, 10–20 µm) were prepared with Al/Ti/C = 5:2:1 (molar ratio) and 4 wt.% CeO_2_ powder (99.0 wt.%, with different average sizes, i.e., 30 nm, 50 nm, 1 μm, 2–4 μm). The mixture was evenly mixed in a PULVERISETTE-5 high-speed planetary ball mill with a ball-to-material ratio = 3:1. The rotation speed and the total milling time were 350 r/min and 3 h, respectively. Then, 50 g of the mixture was cold-pressed into a cylindrical prefabricated block of Φ 25 mm × 50 mm on an AG-10TA universal test stretching machine (Shimadzu Corporation, Kyoto, Japan) and dried in a drying oven at 200 °C for 2 h.

Simultaneously, commercial pure aluminum (99.7 wt.%, A 99.7) was melted in alumina crucible by using a SG-7.5–10 type crucible furnace (Zhonghuan Experimental Furnace Corporation, Tianjin, China). After the temperature was raised to 800 °C, the prefabricated block was added to the melt through the graphite bell jar. According to the previously determined typical sampling time (8, 50, 60, 80 and 90 s), the reaction block was quickly removed at each time point and subjected to high pressure ice brine flow quenching to obtain quenched samples.

The specimens with a size of 10 mm^3^ taken from the quenched samples were electrolytically polished by a reagent (10 vol.% HClO_4_ + 90 vol.% absolute ethanol, voltage is 20 V and the time is about 10~30 s) after mechanical grinding and polishing. The phase constituents of specimens were analyzed using a D8 advance X-ray diffractometer (XRD, Shimadzu Corporation, Kyoto, Japan). The microstructure was examined using a JSM-6700F scanning electron microscope (SEM) equipped with an energy dispersive spectrometer (EDS, Shimadzu Corporation, Kyoto, Japan). In addition, DSC analysis of the Al-Ti-C-Ce system was conducted with a NETZSCH STA 449F3 instrument (NETZSCH, Hanau, Germany) with a heating rate of 20 K/min and samples weighing less than 10 mg.

### 2.2. Grain Refinement Experiments

To evaluate the grain refining effect of Al-Ti-C master alloy after adding CeO_2_, the different master alloys were prepared using the aluminum melt in situ reaction method, and then master alloys were used to refine A 99.7. The specific experimental process is as follows:

Firstly, A 99.7 ingots were melted and heated up to 800 °C in an alumina crucible by using a SG-7.5–10 type crucible furnace (Zhonghuan Experimental Furnace Corporation, Tianjin, China). A proper quality of prefabricated block was then added to the melt after preheating at 200 °C for 2 h. After being held at 800 °C for 15 min, the melt was finally poured into a steel die with an inner diameter of 45 mm, a diameter of 70 mm and a height of 70 mm. By adjusting the size and addition of CeO_2_, Al-Ti-C and Al-Ti-C-Ce master alloys were obtained. The specific experimental parameters and numbers of master alloys are shown in Table 1.

Thereafter, an appropriate amount of A 99.7 was melted in a crucible electrical resistance furnace at about 730 °C. The 0.3 wt.% master alloys were sectioned at the position of 10 mm from the bottom on #1~#5 alloys and then added to the melt. After that, the melt was held for 10 min and finally poured into the steel mold. By solidification, the refined samples were obtained. For comparison, standard A 99.7 was treated in the same process. A specimen with a thickness of 10 mm was cut along the bottom of the refined sample. A cross profile of the specimen was etched using Keller’s reagent (25 vol.% H_2_O + 15 vol.% HNO3 + 15 vol.% HF + 45 vol.% HCl). The grain structures of specimens were taken by a camera, and the average grain sizes were calculated using the linear intercept method.

## 3. Results and Discussion

### 3.1. The Raw Materials of CeO_2_ and the Mixed Particles

**Figure 1:**
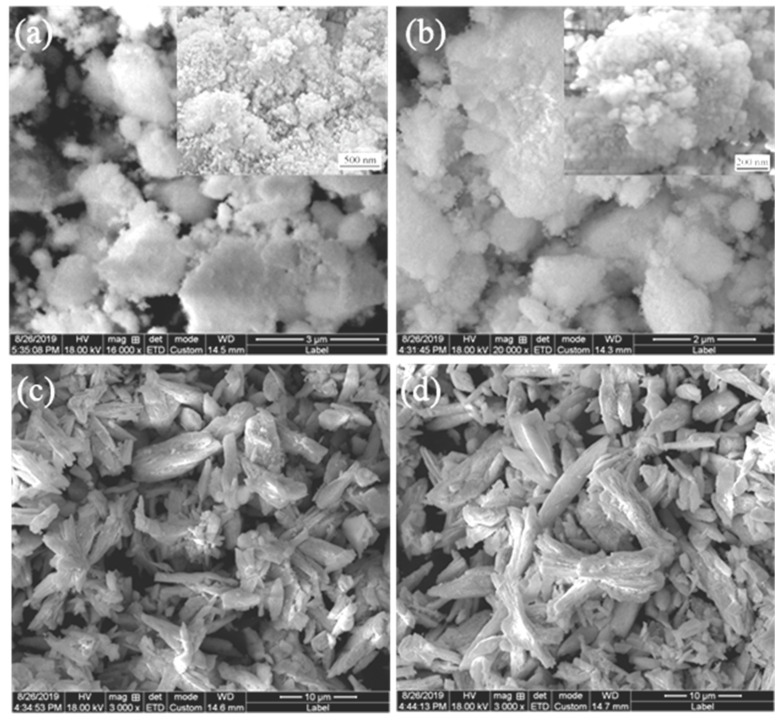
Different sizes of CeO_2_: (**a**) 30nm; (**b**) 50nm; (**c**) 1 μm; (**d**) 2–4 μm.

The raw materials of different CeO_2_ size are presented in Figure 1. From Figure 1a,b, it can be seen that the morphology of CeO_2_ with a size of 30 and 50 nm is spherical nanoparticles. In contrast, the 1 and 2–4 μm sized CeO_2_ gathered together in a short rod shape and small block shape, as shown in Figure 1c,d.

**Figure 2:**
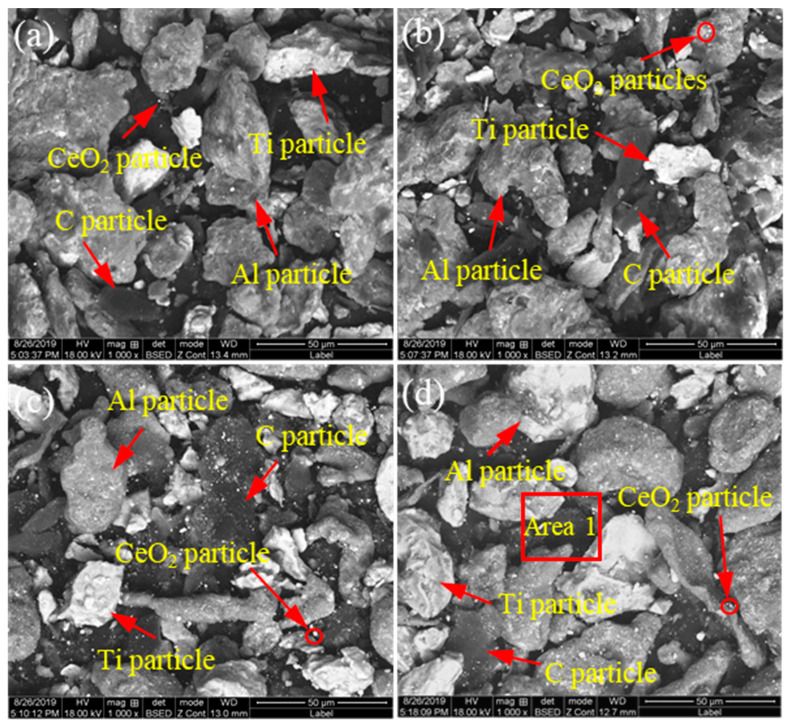
The microstructure of the mixed powders (Al, Ti, C and CeO_2_) with different CeO_2_ size after ball milling: (**a**) 30 nm; (**b**) 50 nm; (**c**) 1 μm; (**d**) 2–4 μm.

**Figure 3:**
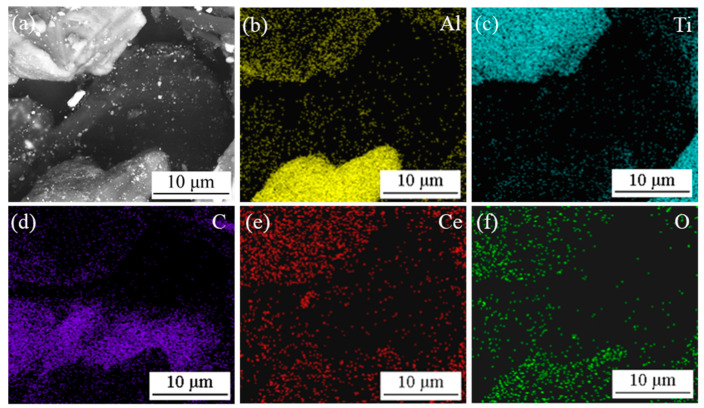
SEM image and map scanning patterns of Area 1 in Figure 2d: (**a**) SEM image; (**b**–**f**) map scanning patterns of Al, Ti, C, Ce and O elements, respectively.

Figure 2 and Figure 3 show the microstructure and EDS results of the mixed powders (Al, Ti, C and CeO_2_) with different CeO_2_ size after ball milling. As shown Figure 2, Al, Ti, C and CeO_2_ powders were evenly mixed after ball milling. Combining the EDS mapping analysis of Al, Ti, C, Ce and O elements as shown in Figure 3, it can be seen that the bright white particles with larger size are Ti, gray particles are Al and black particles are C. The bright white particles with small size are mainly enriched with Ce and O elements, which can be judged as CeO_2_ particles. As can be observed in Figure 2a,b, for nano-CeO_2_ particles, ball milling mainly leads to uniform dispersion. However, for the mixed powder of CeO_2_ with a larger size, ball milling not only contributes to dispersion but also destroys the aggregation between CeO_2_, breaking the CeO_2_ into smaller particles. A statistical analysis of the particle size of CeO_2_ found that after ball milling, the size of 1 μm CeO_2_ particles was reduced to about 400 nm (see Figure 2c), and the size of 2–4 μm CeO_2_ particles was reduced to about 200 nm (see Figure 2d).

### 3.2. Effect of CeO_2_ Particle Size on Phase Transformation and Microstructure Transformation of Al-Ti-C Master Alloy during Preparation

**Figure 4:**
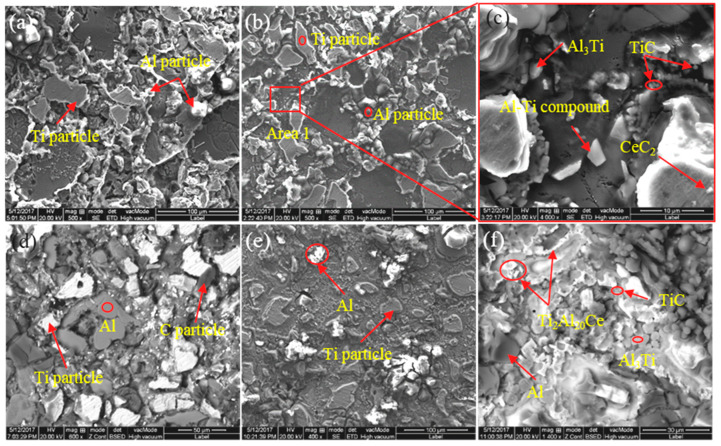
The microstructure of quenching samples with different CeO_2_ size at 60 s: (**a**) 30 nm; (**b**) 50 nm; (**c**) SEM image of Area 1; (**d**) 1 μm; (**e**) low magnification image and (**f**) high magnification image of samples with CeO_2_ of the size of 2–4 μm.

In order to study the influence of CeO_2_ size on the microstructure transformation process of Al-Ti-C system, the microstructure of quenched samples with different CeO_2_ sizes at the same reaction time (60 s) was analyzed. SEM images and EDS results are shown in Figure 4. Comparing Figure 4a,b, it is found that when the CeO_2_ size is 30 and 50 nm, it has little effect on the reaction process of the system. As can be seen, the molten Al closely connects Ti particles, and incomplete Ti particles and incomplete melting Al particles were reserved in system after solidification. As can be seen from Figure 4c, a variety of compounds with different sizes and morphologies are formed around the Ti particles, which are Al_3_Ti, TiC, Al-Ti compounds and CeC_2_ particles. It can be seen from Figure 4d that when the CeO_2_ size is 1 μm, there is no close connection between the Ti particles and the aluminum matrix. Otherwise, compared to Figure 4a,b the degree of reaction is low, and there are obvious incompletely reacted Al, Ti and C particles in the system. When CeO_2_ with a size of 2–4 μm is added to the system, as shown in Figure 4e,f, it can be seen that the size of the Ti particles is reduced, the reaction intensity is enhanced, the density of the system is increased and the particles are closely connected. Especially, A large number of TiC, Al_3_Ti and Ti_2_Al_20_Ce phases are formed in the Al matrix.

This difference in response is because the larger the CeO_2_ particles, the larger the specific surface area and the stronger the catalytic effect, which obviously accelerates the progress of the reaction. For micron-sized CeO_2_ particles, due to the crushing of CeO_2_ particles during ball milling, more active sites are provided, and the larger the particle size, the more severe the degree of crushing, the more active sites and the greater the promotion of the reaction.

**Figure 5:**
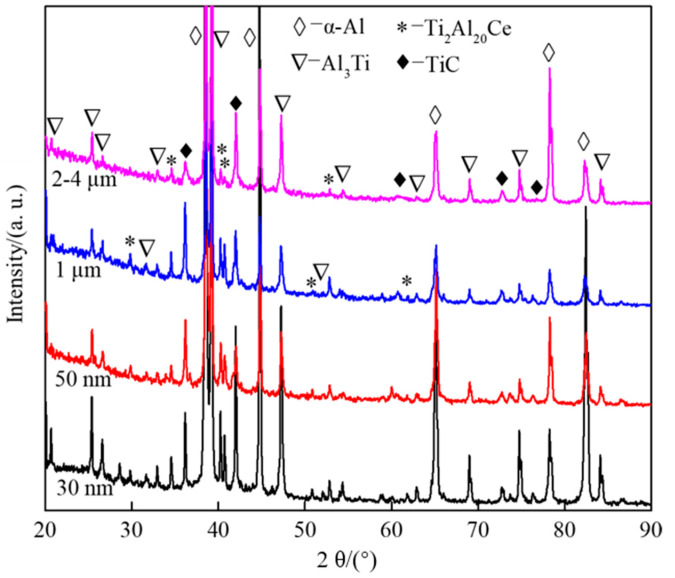
XRD patterns of prefabricated blocks with different CeO_2_ size under complete reaction.

**Figure 6:**
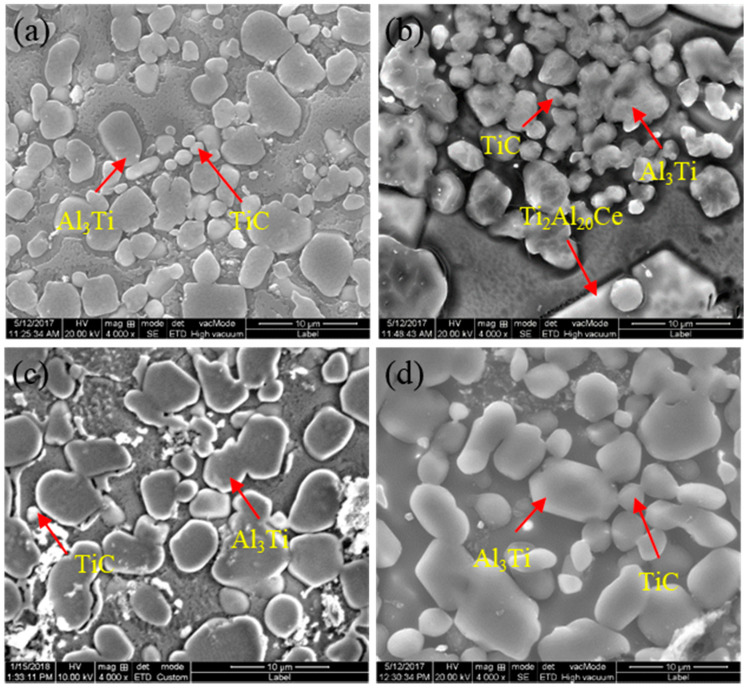
SEM images of the quenching samples of prefabricated block with different CeO_2_ size under complete reaction: (**a**) 30 nm; (**b**) 50 nm; (**c**) 1 μm and (**d**) 2–4 μm.

**Figure 7:**
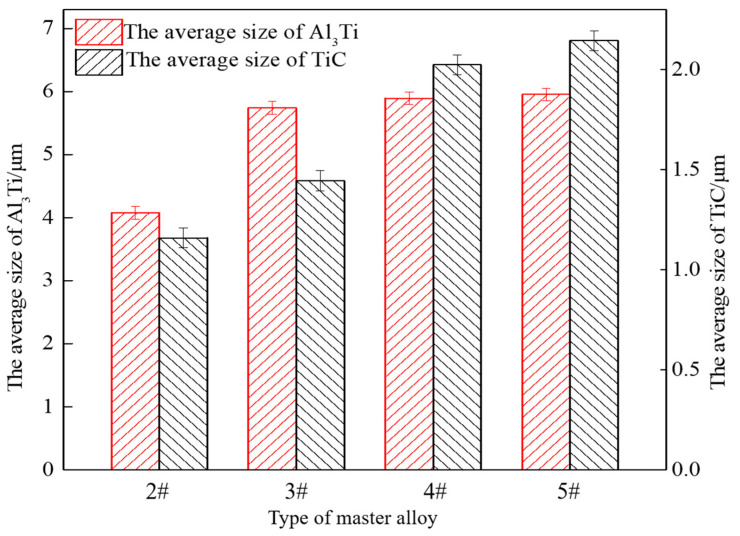
The statistics chart of the second phase particles in prefabricated block with different CeO_2_ size under complete reaction.

The microstructure of the quenched samples with different CeO_2_ sizes added under complete reaction was observed. The phase, microstructure characteristics and the average grain size of the second phase particles of the quenched samples were analyzed, as shown in Figure 5, Figure 6 and Figure 7.

Figure 5 shows XRD patterns of prefabricated blocks with different CeO_2_ size under complete reaction. As can be seen from Figure 5, under complete reaction, the main phases in the sample are α-Al, Al_3_Ti, TiC and Ti_2_Al_20_Ce. When the CeO_2_ particle size is 30 nm and 2–4 μm, the diffraction peak intensity of TiC (2θ = 41.7°) is higher than 50 nm and 1 μm, and the diffraction peak intensity of Al_3_Ti, TiC and Ti_2_Al_20_Ce showed a weakening trend with the increase in CeO_2_ size.

Figure 6 shows SEM images of the quenching samples of prefabricated block with different CeO_2_ size under complete reaction. Comparing Figure 6a–d, it is found that the size of CeO_2_ particles has a great influence on the quantity, morphology and distribution of Al_3_Ti and TiC. When the size of CeO_2_ is 30 nm and 2–4 μm, the amount of TiC and Al_3_Ti is large and the size is relatively uniform, the distribution is dispersed in the matrix. When the size of CeO_2_ is 50 nm, TiC and Al_3_Ti adhere to each other in an irregular shape, and the size is not uniform. When the size is 1 μm, the number of TiC particles is small, and Al_3_Ti is evenly distributed in the matrix.

Figure 7 shows the statistics chart of the second phase particles in prefabricated block with different CeO_2_ size under complete reaction. It can be seen from Figure 7 that the particle size of CeO_2_ mainly affects the size of TiC particles, and has little effect on the size of Al_3_Ti. When the size of CeO_2_ is 30 nm, the average size of Al_3_Ti and TiC is the smallest at 4.0 and 1.1 μm, respectively. With the increase in CeO_2_ particle size, the average size of TiC gradually increases, the average size of Al_3_Ti increases first and then tends to be flat and CeO_2_ particle size is 2–4 μm. The average size of Al_3_Ti in the quenched samples is 6 μm, and the average size of TiC is about 2.2 μm.

**Figure 8:**
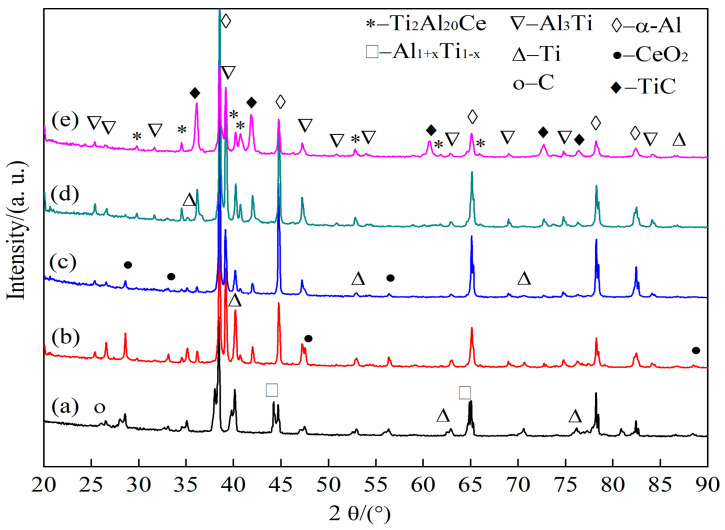
XRD spectra of prefabricated blocks with CeO_2_ particle size of 2–4 μm at different reaction times: (**a**) 8, (**b**) 50, (**c**) 60, (**d**) 80, (**e**) 90 s.

**Figure 9:**
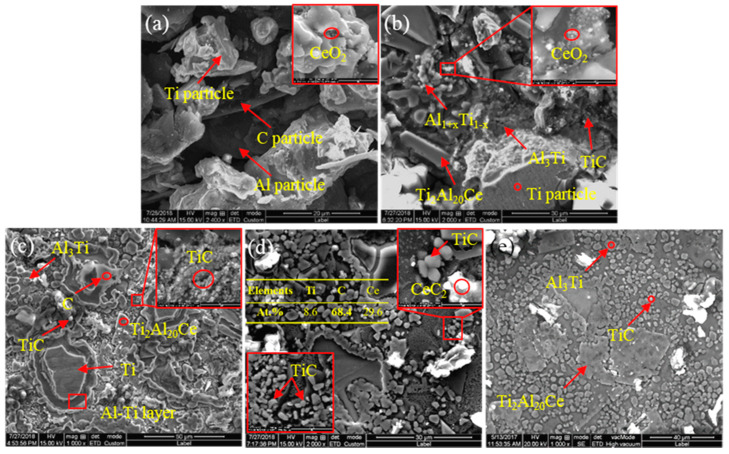
SEM images of #5 quenching samples prepared at different times: (**a**) 8; (**b**) 50; (**c**) 60; (**d**) 80 and (**e**) 90 s.

**Figure 10:**
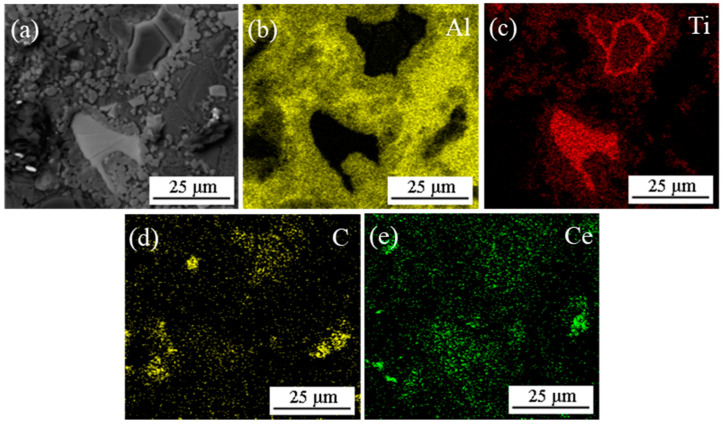
The map scan patterns of Figure 9d: (**a**) backscattered electron image of Figure 9d; (**b**–**e**) map scanning patterns of Al, Ti, C and Ce elements, respectively.

From the above analysis, it can be seen that the content and size of CeO_2_ have a significant effect on the formation, distribution, size, number and morphology of phases in the Al-Ti-C system. In order to further study the formation and transformation process of various phases, the pre-fabricated blocks with CeO_2_ size of 2–4 μm and 4 wt.% were taken as the research objects to study the reaction products and microstructures of the Al-Ti-C-Ce system at different stages. The XRD pattern and microstructure are shown in Figure 8, Figure 9 and Figure 10.

As can be seen from Figure 8a, in the initial stage of the reaction (8 s), the main detection phases in the system are Ti, C, CeO_2_ and a small amount of Al_1+x_Ti_1-x_, indicating that there is a weak solid–solid diffusion process between solid Al and solid Ti at this stage. When the reaction time reaches 50 s, TiC, Al_3_Ti and trace Ti_2_Al_20_Ce are formed, as shown in Figure 8b. As the reaction progresses, the intensity of the diffraction peaks of Al_3_Ti and TiC decreases first and then increases. The intensity of the diffraction peaks of Ti_2_Al_20_Ce continues to increase. When the reaction reaches 90 s, a large amount of TiC, Al_3_Ti and Ti_2_Al_20_Ce are synthesized in the system; only weak incomplete reaction Ti peaks were detected in Figure 8c–e.

As can be seen from Figure 9a, the system basically maintains the original particle state. Figure 9b shows that when the reaction reaches 50 s, the molten Al in the system spreads on the surface of the Ti particles, and a small amount of Al1+xTi1-x is formed under the solid–liquid diffusion reaction [19,20] and then transformed into Ti_2_Al_20_Ce under the action. In addition, around 3 μm Al_3_Ti and 1 μm TiC were formed around the Ti particles, and unreacted Ti particles and CeO_2_ also existed in the system. Figure 9c shows that there is a size of about 5 μm Al3Ti, and bulk Ti2Al20Ce are speculated to be caused by the enrichment of the Al element and the Ce element around the Ti element. In addition, solid C reacts with Ti to form a large number of TiC particles, and the whole system reacts to form a wrapped structure of Ti/Al-Ti/Al_3_Ti/Ti_2_Al_2_0Ce [21,22]. The results of Figure 9d and its surface scanning energy spectrum (see Figure 10) show that rare earth elements are mainly enriched with the surface of C element and around the Ti element, and some rare earth elements react with dissolved Ti atoms and liquid Al to form Al_3_Ti and Ti_2_Al_20_Ce. The other part reacts with solid C to form CeC_2_ and then reacts with dissolved Ti atoms to generate TiC. At the same time, because the affinity between Ti atoms and C atoms is greater than that of Al and C, dissolved Ti atoms directly generate TiC particles with dissolved C atoms. As time goes on, TiC particles grow up, and the encapsulated structure gradually disappears. Al_3_Ti particles are free from the encapsulated structure and freely distributed in the matrix, and the rare earth Ce element continues to enrich around Al3Ti, which hinders the growth of Al_3_Ti particles. The aspect reacts with it to generate Ti_2_Al_20_Ce and grows up by combining [22,23], as shown in Figure 9e. In Figure 9e, TiC particles with an average size of about 2 μm, small bulk Al_3_Ti with an average size of about 6 μm and irregular bulk Ti_2_Al_20_Ce with an average size of about 35 μm were formed in the late stage of the reaction.

**Figure 11:**
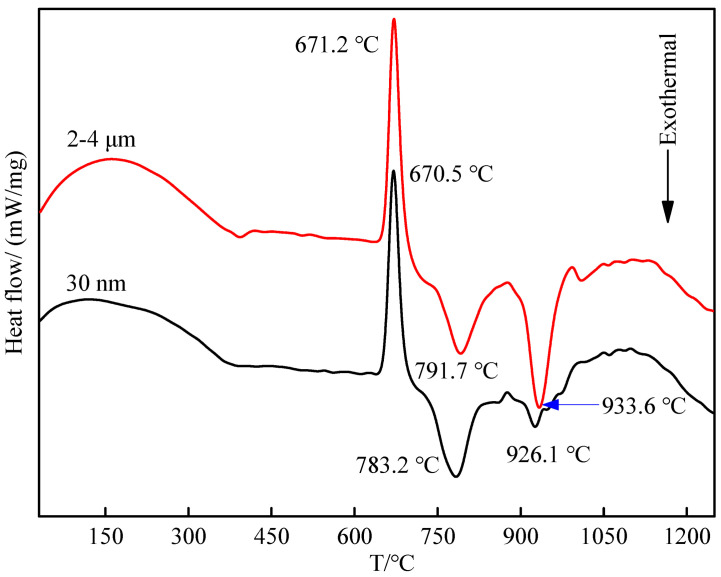
DSC curves of Al-Ti-C-Ce with different CeO_2_ size of: 30 nm and 2–4 μm.

In order to further explore the specific reaction process of the Al-Ti-C-Ce system, the influence of CeO_2_ on the TiC synthesis method of Al-Ti-C system was studied. Combined with the DSC curve of the Al-Ti-C system in the previous research report [24], differential scanning calorimetry analysis was performed on the preforms with CeO_2_ size of 30 nm and 2–4 μm. The DSC curve is shown in Figure 11. The corresponding reaction heat changes are shown in Table 2. From the curve, an obvious endothermic peak and two exothermic peaks can be seen. The first endothermic peaks at temperatures of 670.5 and 671.2 °C represented the melting of aluminum, and the corresponding reaction endotherms were 173.5 and 195.5 J/g. Subsequently, the carbothermal reaction of CeO_2_ with C and oxygen produces CeC_2_, the reaction of Al with Ti produces Al_3_Ti and CeC_2_ reacts with dissolved Ti atoms to produce TiC. These reactions release a lot of heat [14,25]. Combined with literature reports [26,27], in the aluminum-rich melt, Al and Ti first form Al_3_Ti, and the first exothermic peak can be determined as the formation peak of Al_3_Ti. The formation temperatures are 783.2 and 791.7 °C, corresponding to the exothermic heat of 168.8 and 137.7 J/g. The second exothermic peak is the peak formed by TiC, the formation temperatures are 926.1 and 933.6 °C, and the corresponding exotherms of the reaction are 92.99 and 198.6 J/g. Compared with the Al-Ti-C system, it is found that the addition of CeO_2_ does not affect the melting point temperature of aluminum, but it reduces the latent heat of aluminum melting and accelerates the melting of aluminum. The smaller the size of CeO_2_, the lower the heat required for aluminum melting. Furthermore, the addition of CeO_2_ reduces the formation temperature and heat release of Al_3_Ti and TiC. The smaller the size of CeO_2_, the lower the formation temperature. The lower the heat released, the lower the temperature of the system, which affects the growth rate and growth habit of the second phase particles and is beneficial to obtaining Al_3_Ti and TiC with smaller size and regular shape.

### 3.3. Refinement Performance Evaluation

**Figure 12:**
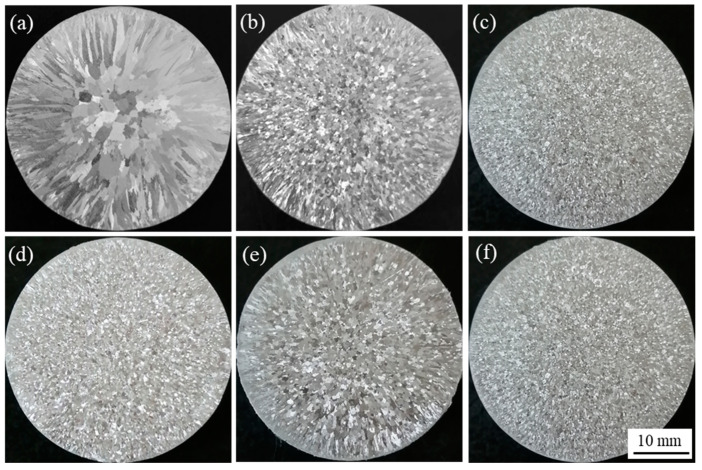
The macrographs of A 99.7 with 0.3% different master alloys: (**a**) unrefined; (**b**) #1; (**c**) #2; (**d**) #3; (**e**) #4; (**f**) #5.

Figure 12 shows the macrographs of A 99.7 with different 0.3% master alloys. Figure 12a indicates that the unrefined pure aluminum is mainly composed of coarse equiaxed crystals in the center and columnar crystals in the middle and edges. From Figure 12b, it can be seen that Al-Ti-C master alloy has a significant refinement effect on A 99.7. After adding it into the melt of A 99.7, the coarse equiaxed crystals are refined into fine equiaxed crystals, while the range of columnar crystals is narrowed and the size is reduced. It can be seen from Figure 12c–f that the refining effect of Al-Ti-C-Ce master alloy is higher than Al-Ti-C master alloy, and the #2 alloy and #5 alloy have a better refining effect than #3 alloy and #4 alloy. Among them, the refining effect of #4 alloy with CeO_2_ size of 1 μm is the worst.

**Figure 13:**
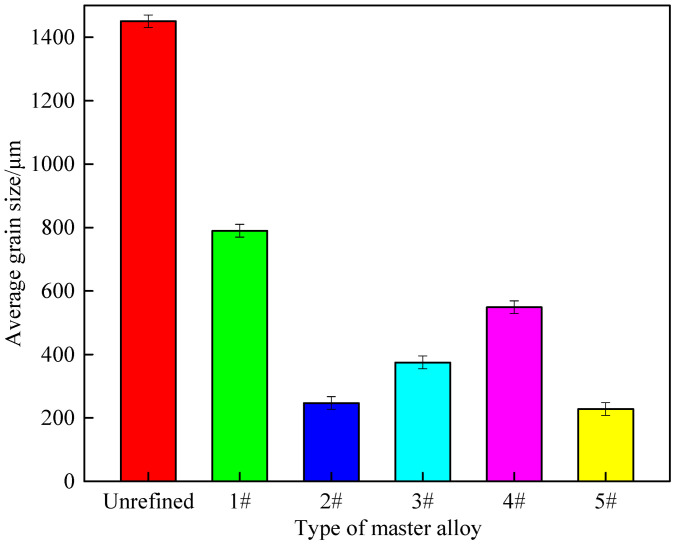
Average grain size of the refining samples in Figure 12.

Figure 13 shows the statistical results of the average grain size of the refined sample in Figure 12. It can be seen from Figure 13 that after 0.3% of Al-5Ti-0.62C, master alloys can refine the A 99.7 grain size from 1430 to 790 μm, while 0.3 wt.% #2 alloy refined the grain size of A 99.7 to 248 μm, and with the increased size of CeO_2_, grain size of A 99.7 tends to increase first and then decrease. The #5 alloy has a significant refinement effect on A 99.7, which can refine the grain size to 227 μm. The above analysis shows that the addition of CeO_2_ with a size of 2–4 μm to master alloy can promote its refining effect.

## 4. Conclusions

(1) The Al-Ti-C-Ce system is mainly composed of α-Al, Al_3_Ti, TiC and Ti_2_Al_20_Ce. The addition of CeO_2_ can obviously speed up the progress of the reaction, and the promotion effect of CeO_2_ size is 2–4 microns.
(2) The addition of CeO_2_ can promote the uniform distribution of Al_3_Ti and TiC, reduce the size of Al_3_Ti and TiC, and the smaller the size of CeO_2_, the smaller the size of Al_3_Ti and TiC synthesized in the system.
(3) The addition of CeO_2_ reduces the latent heat of melting of aluminum, accelerates the melting of aluminum, promotes the reaction process and lowers the formation temperature of second-phase particles. The smaller the size of CeO_2_, the lower the formation temperature of Al_3_Ti and TiC, and the smaller the heat released by the synthesis of TiC.
(4) Al-Ti-C-Ce master alloy has a significant refinement effect on A 99.7. With the increase in CeO_2_ size, the refinement effect tends to increase first and then decrease. When the CeO_2_ size is 2–4 μm, Al-Ti-C-Ce master alloy has the best refining effect.

## Figures and Tables

**Table 1:** The experimental parameters and numbers of master alloys.

Sample No.	Composite of Master Alloy	Size of CeO_2_	Preparation Temperature/°C	Holding Time/Min
#1	Al-Ti-C	None	800	15
#2	Al-Ti-C-Ce	30 nm	800	15
#3	Al-Ti-C-Ce	50 nm	800	15
#4	Al-Ti-C-Ce	1 μm	800	15
#5	Al-Ti-C-Ce	2–4 μm	800	15

**Table 2:** The corresponding heat change of the reaction.

Type of Alloy	△H_Al_/(J/g)	△H_Al3Ti_/(J/g)	△H_TiC_/(J/g)
Al-Ti-C	214.7	−163.4	−838.8
Al-Ti-C-Ce (30 nm)	173.5	−168.8	−92.99
Al-Ti-C-Ce (2–4 μm)	195.5	−137.7	−198.6

## Data Availability

Exclude this statement.
